# Exploring treatment models for oligometastatic castration-sensitive prostate cancer: evolution of therapeutic strategies

**DOI:** 10.3389/fonc.2026.1778687

**Published:** 2026-02-12

**Authors:** Haizhong Xu, Jialin Guo, Wenrui Peng, Jianqiang Wang, Longlong Shi, Xukai Yang

**Affiliations:** 1 Department of Urology, The 940 Hospital of Joint Logistics Support force of Chinese People’s Liberation Army, Lanzhou, China; 2 The First Clinical Medical College, Gansu University of Chinese Medicine, Lanzhou, China

**Keywords:** biomarkers, MDT, multimodal therapy, OmCSPC, PSMA-PET/CT

## Abstract

Oligometastatic castration (hormone)-sensitive prostate cancer (omCSPC) represents an intermediate disease state between localized and widely metastatic cancer. This review synthesizes current evidence on diagnosis, treatment, and biomarkers in omCSPC to guide personalized therapeutic strategies and improve clinical outcomes. A comprehensive literature review was conducted, focusing on the role of PSMA-PET/CT in lesion detection, evolving local and systemic treatment modalities, and the prognostic and predictive value of biomarkers in omCSPC.PSMA-PET/CT has improved the precision of oligometastatic lesion identification. Treatment of omCSPC has evolved from Androgen Deprivation Therapy (ADT) alone to combination regimens incorporating novel hormonal agents or chemotherapy. Local therapies, including radiotherapy and surgery, are increasingly utilized and may delay disease progression. omCSPC exhibits clinical and molecular heterogeneity, with several emerging biomarkers showing prognostic potential, though standardization is still needed. The management of omCSPC requires a multidisciplinary approach and personalized treatment strategies. Further research into biomarkers and the optimal integration of local therapies is essential to improve patient quality of life and long-term survival.

## 1 Introduction

Prostate cancer (PCa) is the second leading cause of cancer-related mortality among males worldwide, a significant proportion of patients present with metastatic disease at the time of their initial assessment, often resulting in a poor prognosis and a paucity of treatment options (1, 2). Androgen deprivation therapy (ADT) is the standard treatment for metastatic castration-sensitive prostate cancer (mCSPC), but resistance limits its efficacy, with most patients developing castration resistance within 24–36 months, facing challenges such as limited treatment options and reduced survival rates (3). How to improve the efficacy of mCSPC and delay progression to metastatic castration-resistant prostate cancer (mCRPC) is a current research focus. Hellman and Weichselbaum initially advanced the oligometastatic theory, which postulates that a nexus state exists between localized lesions and widespread metastatic lesions (4). Oligometastatic castration (hormone)-sensitive prostate cancer (omCSPC) represents the first critical line of defense in advanced PCa, defined by a limited metastatic burden (typically ≤5 metastatic lesions across various imaging modalities) (5–9). This distinctive transitional disease state offers a substantial therapeutic opportunity, as active local intervention has the potential to modify the natural progression of the disease. The 2023 ESMO guidelines now recommend local therapy combined with systemic treatment for omCSPC based on Level 1 evidence, demonstrating overall survival (OS) benefits (10). This paradigm shift reflects our growing understanding of tumor heterogeneity and the limitations of isolated hormonal manipulation. The precise definition of oligometastatic disease (OMD), appropriate treatment sequencing, and the specific role of local therapy in omCSPC remain unresolved, with ongoing debate regarding optimal patient selection, standardized imaging criteria, and key predictive biomarkers. This article integrates current evidence on the management of omCSPC, explores the potential of combined therapy involving primary tumor treatment, metastatic lesion treatment, and systemic therapy in prostate cancer treatment, summarizes biomarkers associated with omCSPC treatment and prognosis, and proposes an evidence-based decision-making framework to provide guidance for the treatment of this clinically unique patient population.

## 2 A new framework for omCSPC beyond lesion counting

The clinical definition of omCSPC remains primarily based on the imaging criterion of ≤5 metastatic lesions (5–9). Nevertheless, this definition, which relies exclusively on the counting of lesions, is unable to capture the significant heterogeneity that exists within this patient population. In order to facilitate the delivery of precise clinical care, it is imperative to establish a multidimensional conceptual framework that considers the imaging-based disease burden, the chronological clinical context, and the genomic biological essence. The integration of these dimensions facilitates the differentiation of biologically distinct subtypes within the “oligometastatic” state, despite similar imaging presentations. Each of these subtypes exhibits markedly different prognoses and treatment strategies.

In relation to the matter of disease burden, current treatment decisions are predominantly informed by the “high/low volume” classification of the CHAARTED criteria. The prospective SABR-COMET-10 trial (NCT03721341) has demonstrated the potential for stereotactic body radiation therapy (SBRT) to be applied to 4–10 metastatic lesions, thereby challenging the conventional “≤5 lesions” threshold and proposing a more flexible approach to treatment boundaries (11, 12). The ESTRO-ASTRO consensus guidelines further emphasise that the feasibility of metastasis-directed therapy (MDT) should take precedence over rigid lesion counting (7). Collectively, these advances support the clinical significance of viewing “oligometastasis” as a continuum, transitioning from minimal burden to early-stage multiple metastases, rather than a binary classification. It is imperative to acknowledge the pivotal role of imaging technology sensitivity in disease burden assessment. A large international multicentre study revealed that patients detected solely by advanced molecular imaging (AMIM) exhibited significantly lower levels of tumour aggressiveness markers (e.g. Gleason score, TP53 mutation rate) and superior 10-year OS (100% vs. 85%) compared to those identified by conventional imaging (CIM) for asynchronous omCSPC (6). This finding suggests that “low burden”, as defined by more sensitive imaging techniques, may more closely represent a biologically inert disease entity.

The temporal context of disease progression constitutes another critical dimension for prognostic stratification. In clinical practice, the timing of metastasis onset (synchronous vs. metachronous) carries fundamental prognostic significance. The OligoCare Consortium classification system provides a core framework for this (7, 8, 13). The fundamental premise of this theoretical framework is the acknowledgement that “asynchronous” recurrence, particularly following protracted treatment-free intervals, may signify a disease with restricted metastatic potential, rendering it more amenable to local curative intervention. Conversely, the presence of ‘synchronous’ disease or “oligo-progression” during systemic therapy is more likely to indicate an aggressive phenotype with established systemic dissemination potential. The clinical data available for consideration provide substantial support for this temporal distinction: survival outcomes for patients with synchronous metastases are significantly worse than those with asynchronous metastases (14). Recent transcriptomic studies have elucidated the molecular basis for this disparity, revealing significantly lower androgen receptor (AR) pathway activity in tumour tissues from synchronous mCSPC (14). Further evidence has been provided which indicates that newly emerging metastases and recurrent metastases following prior local therapy exhibit fundamentally distinct gene expression profiles (15). Collectively, these findings demonstrate that disease timing represents more than a mere clinical classification; it is indicative of underlying differences in tumour biology.

It is imperative to delve into the genomic essence of tumours in order to unravel their heterogeneity. The “disease lineage theory” proposes that metastasis is a continuous process, whereby the mutation frequency of driver genes such as TP53 increases progressively with disease advancement (16). This theory challenges the traditional notion that “oligometastasis” is a fixed entity, suggesting instead that it is more likely a dynamic, heterogeneous disease state. However, a significant limitation of this framework is its reliance on retrospective analysis of late-stage lesions, which fails to provide adequate illumination of molecular events in the early stages of disease evolution. Within this framework, TP53 inactivation serves as a definitive marker of aggressiveness, correlated with rapid progression and high metastatic burden (6, 16). It is noteworthy that TP53 mutations have been observed to correlate with adverse outcomes in cases of clinically considered relatively indolent synchronous omCSPC (6), thereby challenging the reliability of assessing biological behaviour based solely on clinical timing. Conversely, approximately 11% of synchronous omCSPC cases exhibit WNT pathway mutations, which are clearly associated with high visceral metastasis risk and poor prognosis, often coexisting with SPOP mutations (17). This finding suggests that distinct molecular subtypes may be responsible for specific metastatic patterns. This study highlights a critical clinical dilemma: for these patients, it is challenging to determine whether aggressive MDT should be given priority or whether earlier initiation of systemic intensification is more appropriate. This predicament underscores the limitations of conventional imaging-based classification in facilitating precision therapy. Consequently, genomic characteristics are progressively transforming omCSPC from a singular imaging classification into a molecular spectrum encompassing “inert, “ “locally invasive, “ and “systemic-prone” subtypes. The present challenge lies in prospectively validating these molecular markers and integrating them with existing clinical imaging staging to guide treatment decisions. For instance, this could entail the initiation of early intensive systemic therapy for patients deemed to be “systemic-prone”, while concomitantly endeavouring to de-escalate treatment for patients considered to be “inert”. This approach would ultimately result in the realisation of a precision medicine paradigm that is truly driven by biological principles.

The integration of these three dimensions – imaging burden, temporal context and genomic essence – is pivotal to the construction of the omCSPC biology-driven clinical decision framework. The pragmatic management pathway is initiated with a triple assessment: Firstly, it is necessary to clarify the imaging basis of “low burden” (both conventional and molecular imaging), given that their prognostic significance differs markedly. Secondly, it is imperative to ascertain the disease timeline (new concurrent, delayed recurrence, or oligoprogression during treatment) in order to assess its potential aggressive trajectory. It is imperative to emphasise that genomic analysis must identify high-risk molecular signatures (e.g. TP53 or WNT pathway mutations) even when imaging appears consistent with “oligometastasis.” Consequently, the concept of “omCSPC” should be regarded as a continuous disease spectrum encompassing heterogeneous states ranging from “biologically inert low burden” to “biologically aggressive low burden, “ and even “early multiple metastases.” The primary future direction is to integrate multi-level clinical, imaging, and molecular information to refine this cohort, thereby providing a precise roadmap for personalised treatment strategies.

## 3 Current status of diagnostic research on omCSPC

The current diagnostic approach for omCSPC faces significant challenges due to advancements in imaging technology. Traditional imaging methods (bone scan/CT/MRI) often fail to detect metastatic lesions in patients with low PSA levels, while advanced imaging modalities such as prostate-specific membrane antigen (PSMA) positron emission tomography/computed tomography (PET/CT) demonstrate high sensitivity, with a positive detection rate as high as 71.4% even at PSA levels as low as 0.5–1.0 ng/mL (18). PSMA-PET/CT is considered the preferred imaging modality for assessing initial lymph node and distant metastasis in high-risk PCa, enabling precise identification of early metastatic lesions. The aforementioned treatment modality enables early systemic therapy in omCSPC, reduces the necessity for ADT, and accelerates time to ADT resistance (19). In the domain of PET imaging, 18F-sodium fluoride (Na18F) is recognized on a global scale as the preferred radiotracer for identifying osteoblastic metastases, This distinction is attributable to its high affinity for sites of bone remodeling (20). In addition to Na18F, 68Ga-PSMA-11 and 18F-DCFPyL, with their superior targeting properties, represent state-of-the-art imaging modalities tailored for cancer, demonstrating exceptional practicality in identifying oligometastatic PCa lesions (21, 22). PSMA-PET/CT imaging significantly improves the detection rate of bone metastases compared to traditional imaging (e.g., Whole Body Bone Scan, WBBS) (10.3% vs. 7.3%), particularly in the precise localization of fewer metastatic lesions (23). PSMA-PET/CT-guided localized intensified therapy can optimize treatment strategies, reduce the need for systemic therapy, and does not increase the risk of severe adverse events. For example, PSMA-guided radioactive surgery (PSMA-RGS) uses the target-to-background ratio (TBR) ≥ 2 to localize lesions, significantly improving the accuracy of tumor localization and margin assessment, enabling over 50% of cases to avoid extented pelvic lymph node dissection (ePLND) while increasing the detection rate of pathologically positive lymph nodes (24). However, linking imaging findings to long-term outcomes reveals that relying solely on PSMA-PET/CT is insufficient to guide clinical treatment. Kuten et al. (25) found that 15 patients (3.7%) exhibited suspected or highly suspicious bone lesions (27 lesions in total) on PSMA-PET/CT imaging. Based on postoperative Prostate Specific Antigen (PSA) levels and radiological follow-up, 24 of these lesions were ultimately determined to be negative (i.e., false positives), and persistent false-positive bone lesions require follow-up verification. PSMA-PET/CT should be combined with clinical predictive models when guiding lymph node dissection (LND) decisions, rather than used alone (26). The application value of PSMA-PET in efficacy assessment and prognostic prediction is increasingly evident. The limitations of conventional semi-quantitative metrics (e.g., SUVmax) have prompted the development of more comprehensive evaluation systems. Recent evidence indicates that total tumour volume (PSMA-TV) and total lesion uptake (TL-PSMA) derived from baseline PSMA-PET are independent predictors of overall survival in patients with mCRPC (27). Furthermore, parameters describing the spatial distribution of metastases (e.g., DmaxVox) demonstrate clear prognostic significance and can serve as a simplified alternative to PSMA-TV when automated lesion segmentation is unavailable (27). Research frontiers are witnessing a shift towards the utilisation of serial PSMA-PET scans for treatment monitoring. For instance, the prospective PSMA-track trial (NCT06479187) systematically evaluates residual active lesions on PSMA-PET after six months of therapy in patients with mCSPC, exploring its correlation with PSA response. The trial will also analyse multiparametric dynamic changes, including SUV and tumour volume, to inform future treatment intensification or de-escalation based on PSMA-PET results. Collectively, these advances propel PSMA-PET from a static staging tool toward a dynamic management tool capable of participating in prognostic stratification, efficacy assessment, and clinical decision-making. Emerging biomarkers such as polygenic risk scores (PRS) have demonstrated advantages in screening, identifying 71.8% of high-risk cases missed by traditional methods (PSA + MRI) (28). Although PSMA-PET/CT has reshaped the diagnostic and therapeutic framework for oligometastatic disease, its biological heterogeneity requires further exploration, and integration with other testing methods such as liquid biopsy is needed to improve diagnostic accuracy.

## 4 Research on omCSPC treatment

### 4.1 Local treatment

Local therapy has emerged due to the limitations of existing treatment modalities (such as the risk of overtreatment and impact on quality of life (QoL)). Studies have shown that local treatment of the primary tumor in omCSPC can reduce systemic tumor burden, alleviate local symptoms, and enhance sensitivity to radiotherapy (RT) and chemotherapy. Common local treatment modalities include those targeting the primary tumor (RT; cytoreductive radical prostatectomy, cRP) and those targeting metastases (MDT) (29).

#### 4.1.1 Primary lesion treatment

The primary treatment options for mCSPC include RT and cRP, and the potential beneficiary population should be strictly limited to patients with oligometastasis (especially those with ≤3 lesions). In recent years, SBRT has demonstrated significant potential in the domain of prostate cancer treatment (30). With the rapid increase in the number of patients with omCSPC, it has been proposed to combine systemic therapy with local therapies, including RT, to delay progression and improve survival rates by reducing local tumor burden, thereby enhancing oncological outcomes (31, 32). At the Advanced Prostate Cancer Consensus Conference (APCCC), most panel members supported RT for the primary tumor in OMD rather than cRP, and unanimously agreed that systemic therapy should be part of the treatment strategy (33). In accordance with the 2021 European Association of Urology (EAU) guidelines, a combination of ADT and RT is indicated for management of OMD (34). Compared to ADT alone, ADT combined with RT has been shown to improve OS, cancer-specific survival (CSS), progression-free survival (PFS), and delay progression to CRPC in patients with low metastatic burden PCa (35). Compared to RT, cRP is an invasive procedure, but it may offer a viable alternative for patients unwilling to undergo RT. A study evaluating 85 patients with omCSPC whose primary tumors were treated with either RT or RP found no significant differences in 5-year PFS (52.5% vs. 37.9%), CSS (67.6% vs. 84.7%), or OS (63.6% vs. 73.8%) between the RT and RP groups (36). cRP can prolong the time to progression from omCSPC to CRPC, conferring a significant survival advantage to omCSPC patients. Compared with 38 men with cancer of the prostate who received ADT alone, 23 men who received cRP combined with preoperative ADT had significantly longer time without their cancer getting worse (40 months vs. 29 months) and improved time without their cancer getting worse (38.6 months vs. 26.5 months) (37). Rajwa et al. (38) analyzed 116 patients with omCSPC who received cRP treatment, with 69% of patients experiencing no complications and only 11% experiencing grade 2 or higher complications. Cheng et al. (39)looked back at the health records of 1, 733 patients with omCSPC. This study confirmed that combining cRP with ADT improves survival and progression-free survival. It also delays the onset of castration-resistant disease. Currently, several key clinical trials (NCT04461515, NCT04527991, NCT04601390) are evaluating the value of cRP combined with systemic therapy in omCSPC. The EAU has included it as a treatment option for patients with low metastatic burden; however, the optimal treatment regimen (e.g., timing of ADT combination, application of RT) remains undetermined. Prospective trials are needed to validate the efficacy and safety of cRP, balancing surgical complications with survival benefits.

#### 4.1.2 Treatment of metastatic lesions

MDT, represented by SBRT, reduces tumor burden by locally eliminating visible metastatic lesions and demonstrates significant therapeutic value in omCSPC. Studies indicate that systemic-enhanced MDT (SBRT combined with short-course ADT ± androgen receptor pathway inhibitor (ARPI)) improves omCSPC prognosis without compromising QoL and yields superior efficacy compared to monotherapy (40, 41). SBRT can safely delay the initiation of systemic therapy in omCSPC patients and prolong PFS. Results from the STOMP (42, 43) and ORIOLE trials (44) indicate that MDT can delay the initiation of ADT, significantly improve PFS, and induce systemic immune responses. The TRANSFORM trial evaluated the long-term efficacy of SBRT in omCSPC (≤5 lesions), finding that the 5-year treatment-free progression-free survival (TE-FS) rate reached 21.7% (25.4% in the hormone-sensitive subgroup), and the number of lesions (1-3 vs. 4-5) did not affect outcomes, with zero cases of ≥3-grade (G) adverse events (AEs) (45). This study provided the first 5-year evidence-based support for MDT as an alternative to ADT, confirming the universality of MDT for the widely oligometastatic spectrum and highlighting the safety advantages of SBRT. The EXTEND trial confirmed that MDT combined with ADT significantly improved PFS (Hazard Ratio, HR 0.45), radiographic progression-free survival (rPFS) (HR 0.63), and castration-resistant-free survival (CFS) (HR 0.40), and revealed its immune mechanism mediated by T-cell receptor (TCR) repertoire regulation (55% in the combined group vs. 15% in the ADT group) (40). These results show the clinical benefit of MDT combined with ADT. They demonstrate that immune responses in the body (including T cell growth and selection) may be the key biological reason for slowing down disease progression. This provides scientific evidence at the mechanistic level for the “local-systemic” synergistic treatment model for omCSPC. The findings of this study serve to further validate the synergistic value of MDT in combination with systemic therapy, as evidenced by the analysis of real-world data. A multicentre study by Ferriero et al. (46) in chemotherapy-naïve mCRPC patients demonstrated that for those with oligoprogression during abiraterone or enzalutamide treatment, local therapies such as stereotactic radiotherapy targeting progressive lesions significantly prolonged PFS in patients with low tumour burden. This finding aligns with the prevailing clinical strategy, which advocates for the aggressive management of patients who have demonstrated a favourable response to systemic therapy, yet subsequently experience localised progression. The utilisation of MDT in such cases has been shown to effectively control the disease, thereby postponing the necessity for systemic treatment regimen modification. Wang et al. (47) found that omCSPC patients with ATM/BRCA1/2/RB1/TP53 (HiRi) mutations had poorer outcomes (12-month progression rate of 100% vs. 35% in non-mutated patients, HR 5.95), and increased TCR diversity was independently associated with improved PFS (aHR 0.45). The estimated risk of G 2 and G3 AEs with MDT is significantly lower than with ADT, making MDT suitable for omCSPC patients seeking to avoid or delay ADT-related toxicities. Grkovski et al. (48)reported that six patients with positive PSMA expression and mCSPC who received two cycles of [177Lu]Lu-PSMA-617 RLT + SBRT did not experience grade 3 or higher toxicity, indicating the safety of the combination therapy. For omCSPC patients with spinal bone oligometastases, the concept of dose escalation for SBRT was proposed, achieving good local control. In a recent study, 19 patients (79.2%) with low-metastatic or low-progressive disease were treated with ablation doses of 30/40 Gy and a 10-fraction synchronized intensive boost (SIB) regimen. The 1-year and 2-year local control (LC) rates were found to be 90.0 ± 6.7% and 83.3 ± 15.2%, respectively. Additionally, 36.0% of patients reported G1 acute toxicity (49). This aligns with the findings of Deodato et al. (50), who reported good local control rates and toxicity profiles with 12 Gy spinal metastasis SBRT boost to involved and adjacent vertebrae following 25 Gy. More studies are needed to show that MDT is better than other local treatment options, determine the best dose and amount of SBRT, and find the best balance between cost and benefit in treating spinal metastasis. When considering systemic treatment intensification strategies, the choice of treatment sequence is also critical. Furthermore, for patients with high-burden mCSPC, real-world data can also inform clinical decision-making regarding treatment sequencing. Another study by Ferriero et al. (51)demonstrated that in this patient population, first-line use of novel endocrine therapy versus first-line chemotherapy showed no significant difference in progression-free survival or overall survival. This suggests that, even in cases of high disease burden, commencing treatment with newer endocrine therapies – which carry a relatively lower risk of adverse effects – and subsequently transitioning to chemotherapy upon disease progression, constitutes a feasible therapeutic approach. This practice-oriented finding emphasises that in treating omCSPC, the sequencing of local versus systemic intensification should be flexibly tailored to each patient’s specific circumstances. Systemic treatment escalation (STE) is an important milestone in PCa progression. For omCSPC patients, SBRT offers good local control and tolerability, making it an attractive method for delaying STE and preventing its side effects. The study analyzed the oncological outcomes of 119 omCSPC patients after SBRT: the median STE-free survival (STE-FS) was 33.4 months, PFS was 22.7 months, 87 patients (73.1%) had stable or decreasing PSA levels after SBRT, only 1 patient experienced ≥3-grade acute toxicity, and no late toxicity was observed (52, 53). SBRT is a safe and effective treatment for omCSPC and a strategy to delay the use of ADT. The combination of SBRT with systemic therapy may provide synergistic effects, but further clinical trials are needed to validate the survival benefits.

### 4.2 Combined omCSPC therapy strategy balancing reinforcement and downgrading

The treatment objectives for omCSPC necessitate a multifaceted approach that encompasses disease management, quality of life, and treatment toxicity, while concomitantly pursuing survival enhancement. This multidimensional objective is driving clinical exploration of ADT-based combination strategies, with the aim of achieving deeper and longer-lasting responses through the synergistic effects of local and systemic therapies (**Table 1**). Current treatment decisions require the integration of prospective clinical trials with real-world evidence, the latter being crucial for revealing actual efficacy and patterns within the complexities of routine clinical practice.

**Table 1:** Research trials on multimodal combination therapy related to omCSPC.

Author	Trials	Number of metastasis	Treatment (patients)	Control (patients)	Results	Safety
Boevé LMS (54)	HORRAD	≥1†	ADT + EBRT[216]	ADT[216]	mOS: 45 (40.4–49.6) vs 43 (32.6–53.4) months; mPSA-PFS: 15 (11.8–18.2) vs 12 (10.6–13.4) months	−
Parker CC (35)	STAMPEDE	≥1†	ADT+RT[1032]	ADT[1029]	FFS: HR 0.76	Grade ≥3 AEs: 39% vs 38%
Decaestecker K (42), Ost P (43)	STOMP	≤3‡	MDT +AS[31]	AS[31]	mADT-FS: 21 (14–29) vs 13 (12–17) months	Grade 1 AEs: 19% vs –
Armstrong AJ (55), Armstrong AJ (56)	ARCHES	≤5†	Enzalutamide+ADT[224]	PBO+ADT[221]	OS: HR 0.59; rPFS: HR 0.27	Grade 3 AEs: 27% vs 26%; Grade >3 AEs: 21% vs 20%
		>5	Enzalutamide+ADT[220]	PBO+ADT[242]	OS: HR 0.55; rPFS: HR 0.33	Grade 3 AEs: 20% vs 22%; Grade >3 AEs: 14% vs 20%
Saad F (57)	ARANOTE	≥1†	Darolutamide+ADT[446]	PBO+ADT[223]	OS: HR 0.81; rPFS: HR 0.54	Grade 3–4 AEs: 30.8% vs 30.3%; Grade 5 AEs: 4.7% vs 5.4%
Chi KN (58), Chi KN (59)	TITAN	≥1†	Apalutamide+ADT[525]	PBO+ADT[527]	24-month OS: 82.4% vs 73.5%; 24-month rPFS: 68.2% vs 47.5%	Grade 3–4 AEs: 42.4% vs 40.8%
Sweeney CJ (60)	ENZAMET	≥1†	TS+Enzalutamide[563]	TS+NSAA[562]	5-year OS: 67% vs 57%; PSA-PFS: 54% vs 25%; cPFS: 56% vs 28%	Grade 3–4 AEs: 47% vs 33%; Grade 5 AEs: 2% vs 2%
Agarwal N (61)	SWOG-1216	≥1†	ADT+Orteronel[638]	ADT+ Bicalutamide[641]	mOS: 47.6 vs 23.0 months; mPFS: 81.1 vs 70.2 months	Grade 3–4 AEs: 43% vs 14%
Fizazi K (62), Fizazi K (63)	LATITUDE	≥3†	AAP+ADT[597]	PBO+ADT[602]	mOS: NR vs 34.7 months; mrPFS: 33.0 vs 14.8 months	Grade 3–4 AEs: 63% vs 48%; Grade 5 AEs: 28% vs 24%
Kyriakopoulos CE (64)	ECOG3805 CHAARTED	≤3†	ADT+DTX[134]	ADT [143]	mOS: NR	–
		>3	ADT+DTX[263]	ADT [250]	mOS: 51.2 vs 34.4 months	–
Marvaso G (41)	RADIOSA	≤3‡	ADT + SBRT[53]	SBRT[52]	mPFS: 32.2 (22.4–NR) vs 15.1 (12.4–13.4) months	Grade ≥3 AEs: 2% vs 0%
Gravis G (65)	GETUG-AFU 15	≥1†	ADT +DTX[192]	ADT[193]	mOS: 58.9 (50.8–69.1) vs 54.2 (42.2–NR) months	–
Deek MP (66)	−	≤5‡	MDT+ADT[105]	MDT[158]	5-year bPFS: 24% vs 29%; dPFS: 11% vs 19%; cPFS: 41% vs 9%	–
Tang C (40)	EXTEND	≤5†	RP+intermittent ADT[43]	intermittent ADT[44]	mPFS: NR vs 15.8 (13.6–21.2) months	Grade 3 AEs: 7.0% vs 4.5%
Conde-Moreno AJ (67)	SBRT-SG 05	≤3†	ADT + SBRT[67]	−	bRFS/PFS: 1-yr 91%/92%, 2-yr 73.7%/81%, 3-yr 50.6%/67%	Grade ≥3 AEs: None
Fizazi K (68)	PEACE-1	≥1†	ADT+DTX +AA[335]	ADT+DTX[335]	OS: HR 0.75; rPFS: HR 0.50	Grade ≥3 AEs: 63% vs 52%
Hussain M (69)	ARASENS	≤3†	Darolutamide+ADT+DTX [154]	PBO+ADT+DTX [146]	OS: HR 0.68	Grade 3–4 AEs: 70.1% vs 61.1%
		>3	Darolutamide+ADT+DTX [497]	PBO+ADT+DTX [508]	OS: HR 0.69	Grade 3–4 AEs: 64.9% vs 64.2%
Turner PG (70)	ADRRAD	≤3†	ADT +RP+Radium-223[30]	−	mPFS: 20.5 months	Grade 3 AEs: 20%
Nickols NG (71)	SOLAR	≤5†	RT\RP+AAT+SBRT[24]	−	Primary endpoint met: 20/24 (no progression); 3/4 non-responders progressed	Grade ≥3 AEs: 12.5%
Nikitas J (72)	SATURN	≤5‡	ADT+AAT+SBRT[26]	−	PSA <0.05 ng/ml at 6 months: 13/26 patients	Grade 2/3 AEs: 21%/21%

† Synchronous omCSPC.

‡ Metachronous omCSPC.

ADT, androgen deprivation therapy; EBRT, external beam radiation therapy; mOS, median overall survival; mPSA-PFS, median prostate-specific antigen-progression-free survival; RT, Radiotherapy; FFS, Failure-Free Survival; HR, hazard ratio; AEs, adverse events; G1-5, grade1-5; MDT, Metastases-directed therapy; AS, active surveillance; mADT-FS, median ADT-free survival; PBO, placebo; rPFS, radiographic progression-free survival; DTX, Docetaxel; SBRT, stereotactic body radiation therapy; mPFS, median progression-free survival; AAP, abiraterone acetate and prednisone; NR, not reached; bPFS, Biochemical progression-free survival; dPFS, distant progression-free survival; cPFS, combined biochemical progression-free survival; RP, radical prostatectomy; bRFS, biochemical recurrence-free survival; TS, testosterone suppression; NSAA, non-steroidal antiandrogen (bicalutamide, nilutamide, or flutamide).

The fundamental principle of multimodal therapy is the concurrent management of both radiographically visible metastatic lesions and potential microscopic dissemination. The APCCC expert consensus indicates a predominance of opinion in favour of systemic therapy in combination with targeted treatment of metastatic lesions over MDT alone (33). This finding aligns with the prevailing academic consensus on the biological characteristics of omCSPC, which is characterised by dual attributes of local progression and systemic dissemination. It is evident from clinical observations that the implementation of aggressive local therapy targeting the primary prostate tumour (e.g. debulking radical prostatectomy or stereotactic radiotherapy) in combination with ADT can result in a decline of PSA levels to a level that is below the detection threshold in a proportion of patients following testosterone restoration. This finding suggests the potential for the treatment to induce deep biological remission (73, 74). The value of radiotherapy combined with ADT in improving local control and survival outcomes has been confirmed by multiple studies (35, 54). A combined analysis of the STOMP and ORIOLE trials further expanded the beneficiary population for MDT, confirming its applicability to patients with concurrent metastases. Furthermore, it was indicated that molecular characteristics, including DNA damage repair gene mutations, have the potential to serve as biomarkers for predicting MDT efficacy (75). These advances signify a shift in treatment strategies for omCSPC from traditional approaches based on anatomical staging toward precision stratified management integrating clinical and molecular features.Novel endocrine therapies, exemplified by enzalutamide, apalutamide, and darolutamide, when employed in conjunction with ADT, have exhibited marked survival benefits across a wide range of mCSPC populations, including omCSPC (55–60, 76, 77). The advent of these agents has engendered novel therapeutic modalities for the management of omCSPC, namely the substitution or deferral of conventional chemotherapy with highly efficacious, readily accessible oral targeted therapies. However, data from clinical trials, such as ARCHES, also necessitate a period of reflection. Even within subgroups with a limited metastatic burden, survival curves clearly diverge when combination therapy is intensified (55). This observation necessitates a re-evaluation of the prevailing notion that “all omCSPC patients are suitable for treatment de-escalation.” It is imperative to emphasise that the clinical pursuit of “de-escalation” should not be construed as a simplistic reduction in treatment intensity. Rather, it should be conceptualised as a meticulous “risk-adaptive therapy” guided by precise scientific principles. Ferriero et al. provide compelling evidence from real-world data that stereotactic radiotherapy targeting progressive lesions during abiraterone or enzalutamide therapy significantly extends PFS in mCRPC patients who have not received prior chemotherapy, when oligoprogression occurs (46). This finding provides practical justification for implementing “response-driven” de-escalation strategies within dynamic disease management.

In addition to the development of innovative endocrine therapies, the exploration of alternative combination regimens has resulted in substantial insights. The SWOG-1216 trial demonstrated that the addition of the CYP17A1 inhibitor orteronel to ADT resulted in improved progression-free survival, but failed to translate into OS benefit (61). Subsequent analyses of this trial further indicated that baseline bone pain at diagnosis was an independent negative prognostic factor (78), underscoring the importance of incorporating symptom burden into comprehensive risk assessment. Conversely, the LATITUDE trial established abiraterone in combination with prednisone and ADT as the standard of care for high-risk mCSPC, resulting in a significant prolongation of overall survival (62, 63). The patient-reported outcome analysis from this study demonstrated that this regimen not only delayed pain progression but also helped maintain QoL (79), with its long-term efficacy closely correlated with a rapid PSA response early in treatment (80). Collectively, these data illustrate distinct patterns of efficacy and symptom control across different combination strategies. The role of chemotherapy in omCSPC is contingent upon precise identification of the disease burden. The findings of the CHAARTED trial demonstrated that the combination of docetaxel and ADT yielded substantial survival benefits for patients exhibiting high tumour burden. However, this survival advantage was not observed in patients with low tumour burden (64). The long-term follow-up results from the GETUG-AFU 15 trial lend further support to these conclusions and suggest that the routine addition of docetaxel to ADT is not essential for treatment-naïve patients with low-burden concurrent omCSPC, potentially at the expense of short-term QoL (65). These findings serve to reinforce the principle of tailoring treatment strategies based on initial tumour burden, whilst also highlighting the clinical necessity of avoiding unnecessary chemotherapy-related toxicity in patients with low tumour burden.

The existence of real-world retrospective evidence serves to further consolidate the foundation for combined therapy. A comprehensive analysis of 263 patients demonstrated that MDT combined with ADT significantly improved long-term biochemical progression-free survival and distant metastasis-free survival compared to MDT alone, with this advantage being particularly pronounced in patients with late-onset metastases (66). For patients with recurrent oligometastatic disease, SBRT combined with ADT has demonstrated favourable local control rates and metastasis-free survival with manageable toxicity, offering a viable treatment option for this population (67).

The ongoing exploration of intensified treatment strategies has continued to extend the boundaries of therapeutic benefit. The PEACE-1 and ARASENS trials established the triple combination of ADT with docetaxel and a novel endocrine agent (abiraterone or darolutamide) as the new standard for high tumour burden/high-risk mCSPC (68, 69). It is noteworthy that in patients with concurrent omCSPC, the integration of radiotherapy into such intensified systemic regimens (e.g., ADT + abiraterone) further extends progression-free survival and castration-resistant survival (81). The findings from network meta-analyses provide further evidence for the survival benefit of triple therapy in these patients (82, 83). However, when applying this current “ceiling” level of intensive therapy to omCSPC, particularly in patients with low tumour burden, it is essential to carefully evaluate whether the additional absolute benefit sufficiently outweighs the significantly increased treatment toxicity, complexity, and healthcare costs. In this context, real-world evidence assumes a pivotal role, offering invaluable insights by illuminating the actual tolerability, completion rates, and long-term impact on quality of life associated with intensive therapies across more heterogeneous patient populations. This, in turn, facilitates the delineation of their optimal boundaries within routine clinical practice. Moreover, pioneering studies are investigating more innovative combinations, such as the integration of ADT, Radium-223, and pelvic radiotherapy. Preliminary results have confirmed the safety of the treatment and demonstrated encouraging signs of efficacy (70). The RADICALS-HD trial’s findings on the optimal duration of adjuvant ADT (84) further underscore the imperative that treatment intensity must be precisely matched to individual recurrence risk.

Consequently, the prevailing emphasis in omCSPC combination therapy is undergoing a gradual transition from the pursuit of a “universal optimal combination” to the exploration of “personalised intensity and timing”. A plethora of studies have indicated the direction in which future research should be directed. The RADIOSA trial demonstrated that for concurrent omCSPC, SBRT combined with short-term (6-month) ADT outperformed SBRT alone (41). The EXTEND trial demonstrated that MDT combined with intermittent ADT not only effectively controls disease but also improves patient QoL by preserving testosterone levels (40). The SOLAR and SATURN trials further validated that short-term (6 months) intensive novel endocrine therapy combined with local curative treatment can achieve deep remission in some patients with newly diagnosed omCSPC, with sustained response after treatment completion (71, 72). Collectively, these studies suggest that for patients who have not yet received treatment, early adoption of short-term intensive therapy combined with local intervention may represent a promising pathway. Nonetheless, treatment decisions for recurrent patients require greater caution and should be made after comprehensive consideration of multiple factors, including prior treatment history (85). The following strategies are predicated on a shared logic: The induction process is characterised by a time-limited period of high intensity, which is followed by a step-down maintenance phase. The objective is to attain optimal disease control benefit with the most concise intensive treatment duration, thereby minimising cumulative toxicity from prolonged therapy. In the future, it is essential for prospective clinical trials and large-scale real-world studies to collaborate in order to validate the broad applicability of these strategies, identify optimal patient populations, and ultimately establish personalised decision models based on dynamic monitoring tools such as PSMA-PET and ctDNA.

In summary, the treatment strategy for omCSPC is shifting towards a precision management framework that integrates dynamic biological behaviour with individualised needs. Decision-making processes should no longer be confined to initial lesion count and type, but must integrate multidimensional information throughout the treatment journey. This should include molecular characteristics, dynamic responses (e.g. PSA kinetics, imaging changes) and patient-reported outcomes. This necessitates the seamless integration of prospective efficacy evidence from randomised controlled trials with practical insights from real-world studies on treatment patterns, tolerability, adherence, and cost-effectiveness, with the objective of collectively informing clinical choices. It is anticipated that future exploration will be conducted across a range of dimensions. In the field of treatment modalities, neoadjuvant chemoradiation combined with robotic surgery and other local therapies has demonstrated potential (86), while emerging strategies such as immune combination therapies are under active investigation (87). The results from prospective trials, such as PERSIAN (NCT05717660), will directly validate the efficacy of the “systemic-local” model combining novel endocrine agents with SBRT (88). The ultimate objective is to establish a dual-track evidence generation system driven by both prospective trials and real-world data. This system will chart a comprehensive, precision pathway for patients – from initial induction therapy to long-term management – fundamentally shifting the medical model from “population-based guidelines” to “individualised care”.

## 5 Treatment-related biomarkers

In the context of precision diagnosis and treatment of prostate cancer, biomarkers that are capable of reliably predicting treatment efficacy and guiding clinical decision-making hold core value. At present, PSA kinetics, novel imaging technologies such as PSMA-PET, and various genomic tests collectively form the biomarker system for personalised management of mCSPC (**Table 2**). Nevertheless, the principal challenges in this domain are not to be found in the number of biomarkers, but rather in the uneven levels of evidence across different categories of biomarkers, disparities in clinical accessibility, and the lack of a unified framework for integrated application. A significant proportion of biomarkers are derived from retrospective data, and their predictive efficacy in complex real-world clinical settings requires further validation. The objective of this section is to methodically categorise existing major biomarkers, with a particular emphasis on evaluating their clinical evidence strength and translational maturity. The ultimate aim is to provide more actionable reference guidelines for clinical practice.

**Table 2:** Research on predictive markers related to the treatment of Mcspc.

Author	Year	Markers	Result	Conclusion
Li X (89)	2023	PSAPDR	PSA decline rate: 11.6 ± 1.5% vs 2.9 ± 2.2% per day (RARP+ADT vs ADT)	A higher PSA decline rate is associated with better treatment response.
Saad F (90)	2024	undetectable PSA (<0.2 ng/ml)	Undetectable PSA (<0.2 ng/mL) rate in low-volume disease: 84% vs 38% (Darolutamide+ADT+DTX vs Placebo+ADT+DTX)	Achieving undetectable PSA with intensified therapy correlates with superior clinical outcomes.
Harshman LC (91)	2018	PSA ≤ 0.2 ng/mL at 7 months	mOS from 7-month PSA: 60.4 months for <0.2 ng/mL vs 22.2 months for <0.4 ng/mL. PSA <0.2 ng/mL rate: 45.3% vs 28.8% (Darolutamide+ADT vs ADT).	PSA <0.2 ng/mL at 7 months is a surrogate for long-term survival; intensified therapy increases its attainment.
Ali A (92)	2021	Bone Metastatic Burden	OS/PFS by metastatic burden: HR 0.62/0.57 (low) vs HR 1.08/0.87 (high)	Prostate radiotherapy improves survival in patients with low metastatic burden (M1a/oligometastatic bone).
Jiménez N (93)	2024	TSGs: RB1, PTEN, and TP53	CRPC-FS & OS: TSGlow associated with shorter survival (HR 1.8/2.0). TSGWT patients on ADT+darolutamide showed significant benefit (HR 0.4).	TSGlow is a prognostic biomarker for poor outcomes in mCSPC.
Hearn JWD (94)	2020	HSD3B1 Genotype	2-yr CRPC-free rate: 51.0% vs 70.5%; 5-yr OS: 57.5% vs 70.8% (HSD3B1 adrenal-permissive vs restrictive genotype)	The HSD3B1 adrenal-permissive genotype is prognostic for faster progression and shorter survival.
Hamid AA (95)	2021	gene expression profile	Benefit from ADT+darolutamide: HR 0.45 (Luminal B subtype) vs HR 0.85 (Basal subtype). Greater benefit with high Decipher risk (HR 0.41).	Molecular subtyping can predict differential benefit from treatment intensification.
Ross AE (96)	2024	Decipher genomic classifier、 AR-Ascore、PAM50 cell subtype signature	Higher Decipher/AR-A scores & luminal subtype associated with: >25% PSA rise on enzalutamide; lower rate of negative biopsy at 2 years.	These molecular features may serve as predictive biomarkers for enzalutamide efficacy.
Kohli M (97)	2020	ctDNA	Higher baseline ctDNA level associated with faster ADT failure (HR 2.29).	ctDNA level is a prognostic marker for time to ADT failure.
Du X (98)	2023	ctDNA sequencing\HRR gene	Median time to event: ctDNA decline (17.70 months) vs rise (8.43 months); with HRR alteration (8.02 months) vs without (13.20 months).	ctDNA dynamics and HRR alterations are indicative of treatment benefit or disease progression.
Andrews JR (99)	2025	PSMA+EVs	bPFS/rPFS: 26.1/36.0 months (low PSMA+EVs) vs 15.0/25.0 months (high PSMA+EVs)	A low PSMA+EVs score is a predictive biomarker for benefit from SABR.
Tang C (40)	2023	TCR regulation	Controlled TCR repertoire rate: 55% vs 15% (MDT+ADT vs ADT). Associated with PFS benefit (HR 0.21).	TCR repertoire modulation predicts benefit from MDT.
Lin HM (100)	2025	PCPro	PCPro-negative status associated with worse OS (HR 1.81) and clinical PFS (HR 1.65).	PCPro status is a prognostic biomarker, supporting investigation of lipid metabolism-targeting therapies.
Zaffaroni M (101)	2023	Nutritional and Inflammatory Status	No significant differences in nutritional/inflammatory scores between groups over time.	Common nutritional/inflammatory parameters lack prognostic utility in this setting.
Wang JH (102)	2025	ArteraAI MMAI	OS: MMAI-high vs MMAI-low (HR = 6.46); TTCRPC: MMAI-high vs MMAI-low (HR = 2.07)	High MMAI score is a strong prognostic marker for poor survival and rapid progression to CRPC.

PSAPDR, prostate-specific antigen percentage decline rate; RARP, robot-assisted radical prostatectomy; ADT, androgen deprivation therapy; DTX, Docetaxel; mOS, median overall survival; PSMA, prostate-specific membrane antigen; EVs, extracellular vesicles; bPFS, Biochemical progression-free survival; rPFS, radiographic progression-free survival; SABR, Stereotactic ablative body radiotherapy; HR, hazard ratio; TSG, tumor suppressor genes; CRPC-FS, castration-resistant prostate cancer (CRPC)-free survival; mCSPC, metastatic castration-sensitive prostate cancer; AR-A, androgen receptor activity; ctDNA, circulating tumor DNA; HRR, homologous recombination repair; MMAI, multimodal artificial intelligence; TTCRPC, time to castration-resistant prostate cancer; clinPFS, clinical progression-free survival; TCR, T-cell receptor; MDT, Metastases-directed therapy.

### 5.1 PSA kinetic parameters

The protein known as PSA has the longest documented history of clinical application of any tumour marker. It is primarily valued for its ease of detection and its suitability for long-term, dynamic monitoring. A plethora of studies have repeatedly demonstrated that achieving deep PSA suppression in the early stages of treatment (e.g., PSA decline to <0.2 ng/mL) is evidently associated with enhanced long-term survival in patients (89–91). This is indicative of the high tumour sensitivity to initial endocrine therapy, which serves as a “post-treatment responsiveness” prognostic indicator. This high-level evidence has become a cornerstone for clinical efficacy assessment. Nevertheless, PSA’s limitations are evident in its inability to predict tumour heterogeneity prior to treatment, a shortcoming that has prompted researchers to continually seek novel, more predictive biomarkers.

### 5.2 Imaging-derived biomarkers

The application of novel molecular imaging techniques, such as PSMA-PET, with their ultra-high detection sensitivity, is profoundly reshaping our understanding of staging in “oligometastatic” disease. The value of these technologies lies not only in more precise lesion detection but also in establishing critical clinical treatment selection criteria. For instance, an analysis of STAMPEDE trial data indicates that survival benefits from prostate radiotherapy are strictly confined to patients with a limited number of bone metastases (≤3) (92). This provides robust, high-level evidence supporting the identification of patient subgroups most likely to derive additional benefit from local therapy, complementing systemic treatment. At present, the assessment of lesion burden and distribution through imaging has become a pivotal reference point for clinical decision-making, with relatively mature applications. However, further research is required to quantify imaging metabolic parameters and establish them as standardised prognostic indicators.

### 5.3 Tumor genomic markers

Genomic markers have been shown to directly reveal the intrinsic biological drivers of tumourigenesis and progression. For instance, the functional inactivation of key tumour suppressor genes (e.g. RB1, PTEN, TP53) has been demonstrated to correlate with highly invasive phenotypes (93, 103, 104), while specific variants in the HSD3B1 gene have been shown to be associated with the rapid development of resistance to ADT in patients (94). Furthermore, molecular subtyping and risk scoring systems based on multi-gene expression profiles (e.g., Decipher) provide more refined prognostic stratification and may indicate differential responses to intensive therapy or specific novel endocrine agents across subtypes (95, 96, 105). The preponderance of evidence for these markers is derived from retrospective translational analyses of substantial clinical trials, yielding moderate-level evidence. While there is clear value in using these tools to predict the course of the disease, more research is needed to understand how they can be used to guide the choice of first-line treatment. A number of practical challenges must be surmounted if widespread clinical adoption is to be achieved. These include the feasibility of obtaining metastatic tissue samples, the cost of testing, and turnaround times.

### 5.4 Liquid biopsy for dynamic monitoring

The analysis of circulating tumour DNA (ctDNA) represents a revolutionary approach to dynamic monitoring. It facilitates non-invasive acquisition of the tumour’s genomic landscape prior to treatment, thereby providing real-time insights into the evolutionary dynamics of tumour clones during therapy. Research has demonstrated that the rapid clearance of ctDNA is indicative of a favourable treatment response. Furthermore, the emergence of specific mutations during treatment, such as homologous recombination repair gene mutations, may signal the onset of resistance at an earlier stage than clinical or imaging progression (97, 98). In the context of CRPC, the predictive value of ctDNA assays, such as AR-V7, is supported by substantial evidence (106, 107). In the mCSPC phase, ctDNA demonstrates significant potential for early treatment response assessment and resistance prediction, with rapidly accumulating evidence. However, high-level studies capable of prospectively guiding initial treatment decisions remain lacking, and standardisation of detection technologies remains a critical issue to address.

### 5.5 Emerging and exploratory biomarkers

This category comprises a multitude of biomarkers that are currently in the early stages of research. For instance, a scoring system based on prostate-specific membrane antigen-positive extracellular vesicles (PSMA+EVs) aims to identify pre-treatment biological subpopulations likely to respond durably to local radiotherapy (99). Dynamic changes in the TCR repertoire (40, 47) and plasma lipidomics profiles (100) offer novel perspectives on disease understanding through immune response and metabolic reprogramming, respectively. It is important to note that “negative results” in biomarker research also hold significant value. For instance, the failure of nutritional inflammation markers to predict treatment outcomes in the RADIOSA trial (101) suggests that generalized systemic inflammatory nutritional indicators may be less effective than tumour-specific biomarkers in the specific context of omCSPC. Despite the evident potential of artificial intelligence models in the integration of multimodal data for prognostic prediction, their “black box” nature necessitates rigorous external validation before clinical implementation (102). In conclusion, the evidence supporting these emerging biomarkers is currently limited, with the current body of research primarily serving to expand scientific understanding. The path to true clinical translation remains arduous.

As our understanding of tumour genomics deepens, the concept of “oligometastasis” is evolving from a radiological phenomenon to one that acknowledges its intrinsic molecular drivers. Specific genetic alterations have been shown to correlate closely with distinct disease progression trajectories (e.g., co-mutations of TP53 and RB1 often predict rapid, extensive progression, while SPOP mutations are frequently associated with indolent disease courses) (17, 108–110). This prompts us to consider that future definitions and stratifications of oligometastasis may need to emphasise molecular subtypes rather than relying solely on the number of lesions. Despite the expansion of the biomarker landscape for mCSPC, the integration of these dispersed discoveries into a decision-making system applicable to clinical practice remains a core challenge. This is due to fragmented evidence and a lack of prospective validation. It is imperative that future research places a premium on the prospective clinical validation of key biomarkers and proactively explores pathways for integrating multi-omics data. This will establish a robust foundation for the construction of a personalised treatment framework that is truly centred on tumour biological characteristics.

## 6 Clinical decision-making framework concept

**Figure 1:**
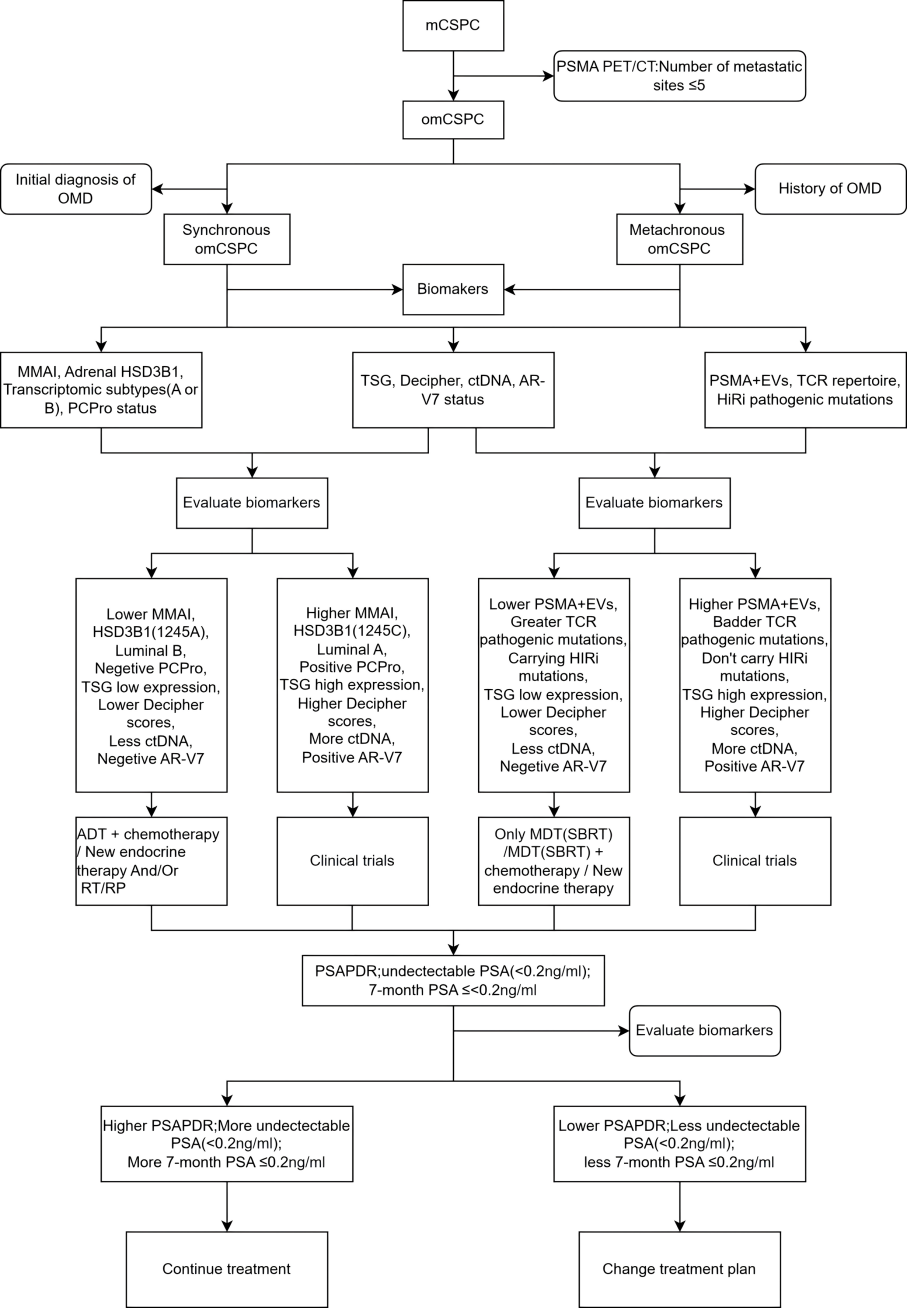
omCSPC clinical treatment decision concept. mCSPC, metastatic castration-sensitive prostate cancer; PSMA PET/CT, prostate-specific membrane antigen (PSMA) positron emission tomography/computed tomography (PET/CT); omCSPC, oligometastatic castration-sensitive prostate cancer; OMD, oligometastatic disease; MMAI, multimodal artificial intelligence; TSG, tumor suppressor genes; ctDNA, circulating tumor DNA; AR-V7, androgen receptor (AR)-V7; PSMA, prostate-specific membrane antigen; EVs, extracellular vesicles; TCR, T-cell receptor; ADT, androgen deprivation therapy; RT, Radiotherapy; RP, radical prostatectomy; MDT, Metastases-directed therapy; SBRT, stereotactic body radiation therapy; PSAPDR, prostate-specific antigen percentage decline rate.

The present study will focus on prostate cancer patients with clear evidence of metastasis who have not received endocrine therapy. According to the findings of PSMA-PET/CT scans, patients with a maximum of five metastatic lesions are to be designated as omCSPC. Subsequent categorization of these patients into synchronous (initial diagnosis with ongoing treatment) and asynchronous (oligorecurrent after treatment) groups is recommended, with this categorization based on the patients’ treatment timeline. The assessment of relevant biomarkers, the screening of patient populations based on biomarker expression, and the selection of treatment regimens with better efficacy are critical (**Figure 1**).

For synchronous omCSPC: Patients with lower levels of multimodal artificial intelligence (MMAI), high-sensitivity C-reactive protein (HSD3B1 [1245A]), luminal B, Negetive PCPro, TSG low expression, lower Decipher scores, less ctDNA, and negative AR-V7 may be treated with ADT + chemotherapy/new endocrine therapy and/or radiation therapy/prostate removal surgery (RT/RP). Patients exhibiting elevated levels of MMAI, HSD3B1 (1245C), Luminal A, positive PCPro, high TSG expression, elevated Decipher scores, increased ctDNA, and positive AR-V7 may be amenable to novel therapeutic regimens currently under investigation in clinical trials. For metachronous omCSPC: The following factors may be indicative of a favorable response to treatment: lower PSMA+EVs, greater TCR pathogenic mutations, carrying HIRi mutations, low TSG expression, lower Decipher scores, less ctDNA, and negative AR-V7 patients. In such cases, treatment may be administered in the following manner: MDT (SBRT)/MDT (SBRT) + chemotherapy/new endocrine therapy treatment regimens.Patients exhibiting elevated levels of PSMA+EVs, more severe TCR pathogenic mutations, an absence of HIRi mutations, heightened TSG expression, elevated Decipher scores, increased ctDNA, and a positive AR-V7 status may qualify for novel treatment regimens currently being evaluated in clinical trials.

The efficacy of the treatment is assessed based on a variety of PSA-related indicators, including the PSADR, the undetectable PSA level (defined as a level of less than 0.2 ng/ml), and the 7-month PSA level, which should be no higher than 0.2 ng/ml. In the event that patients exhibit a higher PSADR, a greater proportion of undetectable PSA (<0.2 ng/ml), and a higher proportion of 7-month PSA ≤0.2 ng/ml, it is advisable to maintain the current treatment regimen. Conversely, if patients demonstrate a lower PSADR, a reduced proportion of undetectable PSA (<0.2 ng/ml), and a lower proportion of 7-month PSA ≤0.2 ng/ml, it is recommended to transition to an alternative treatment regimen.

## 7 Conclusion

Treatment for omCSPC has evolved from a model of solely systemic control to a precision model integrating local therapy for both primary and metastatic sites. The current high-level evidence indicates that for low-burden disease, primary site radiotherapy combined with short-term ADT improves survival. For metastatic disease, targeted therapy (MDT) safely delays escalation of systemic treatment. Meanwhile, novel endocrine therapies combined with ADT form the cornerstone of systemic treatment, with triple therapy emerging as the new standard of care for intensification in patients with high tumour burden. The achievement of a deep PSA response (e.g. PSA <0.2 ng/mL) in the early stages of treatment has become a key prognostic indicator for identifying patients who are eligible for potential downgrading of therapy.

However, translating these clinical trial principles into heterogeneous clinical practice requires real-world evidence (RWE). For instance, local consolidation therapy targeting progressive lesions during systemic treatment has been demonstrated to prolong disease control, thus supporting a dynamic MDT strategy (46). For patients with high-burden disease, initial use of novel endocrine therapy has been shown to yield comparable survival outcomes to chemotherapy, offering clinical flexibility in balancing efficacy and toxicity (51). Moreover, recent studies have revealed disease heterogeneity masked by traditional classifications. A 2025 Japanese multicentre study offers novel insights into the contemporary management of endocrine therapies in patients with lung metastases. The study demonstrates that these patients demonstrate favourable prognosis and significantly better survival than those with other visceral metastases. Furthermore, the presence of bone metastasis has been shown to be a more significant prognostic factor than the status of lung metastasis (111). This finding suggests that the binary assumption in the CHAARTED criteria, whereby visceral metastasis is inherently equated to a poor prognosis, may be overly simplistic in actual clinical settings. Concurrently, the RADIOSA trial confirmed that for synchronous oligometastases, SBRT combined with only 6 months of short-term ADT significantly extended median PFS to 32.2 months with good tolerability (41). This finding directly validates the feasibility of the “time-limited high-intensity induction” strategy in balancing efficacy and toxicity. Consequently, the precision management of omCSPC must integrate “efficacy” evidence from randomised controlled trials (RCTs) with “effectiveness” and “feasibility” data from real-world studies.

In the context of future projections, there are several pivotal areas that necessitate urgent exploration. The primary task is to prospectively validate the predictive value of biomarkers, such as molecular subtyping and ctDNA dynamics, for treatment intensification or de-escalation. Secondly, it is essential to clarify the long-term efficacy and optimal patient populations for strategies such as “time-limited intensification followed by maintenance de-escalation, “ while optimising the combination and timing of multidisciplinary teams (MDTs) with different systemic treatment regimens (particularly triple therapy). The ultimate objective is to construct a viable clinical decision model integrating clinical staging, multi-omics biomarkers, and dynamic assessment tools such as PSMA-PET and PSA kinetics to enable real-time interpretation of disease biology and personalised navigation.

Collectively, these efforts point towards a fundamental shift in the management framework for omCSPC. The transition is from a static staging model based on anatomical lesion counts to a precision classification model grounded in dynamic biological behaviour. Consequently, the subsequent management system will be the synergistic consequence of high-level clinical trial evidence, structured real-world data, and dynamic multidimensional biomarkers. The objective of this pathway is to transform the current anatomical staging framework into a system that is truly biology-driven and precision treatment-oriented.

## References

[1] Sung H Ferlay J Siegel RL Mathieu L Isabelle S Ahmedin J . Global cancer statistics 2020: GLOBOCAN estimates of incidence and mortality worldwide for 36 cancers in 185 countries. CA Cancer J Clin. (2021) 71:209–49. doi: 10.3322/caac.21660, PMID: 33538338

[2] Culp MB Soerjomataram I Efstathiou JA Bray F Jemal A . Recent global patterns in prostate cancer incidence and mortality rates. Eur Urol. (2020) 77:38–52. doi: 10.1016/j.eururo.2019.08.005, PMID: 31493960

[3] Lorente D De Bono JS . Molecular alterations and emerging targets in castration resistant prostate cancer. Eur J Cancer. (2014) 50:753–64. doi: 10.1016/j.ejca.2013.12.004, PMID: 24418724

[4] Hellman S Weichselbaum RR . Oligometastases. J Clin Oncol. (1995) 13:8–10. doi: 10.1200/JCO.1995.13.1.8, PMID: 7799047

[5] Dingemans AC Hendriks LEL Berghmans T Levy A Hasan B Faivre-Finn C . Definition of synchronous oligometastatic non-small cell lung cancer-A consensus report. J Thorac Oncol. (2019) 14:2109–19. doi: 10.1016/j.jtho.2019.07.025, PMID: 31398540

[6] Sutera P Song Y van der Eecken K Shetty AC English K Hodges T . Clinical and genomic differences between advanced molecular imaging-detected and conventional imaging-detected metachronous oligometastatic castration-sensitive prostate cancer. Eur Urol. (2023) 84:531–5. doi: 10.1016/j.eururo.2023.04.025, PMID: 37173210 PMC10636237

[7] Nevens D Jongen A Kindts I Billiet C Deseyne P Joye I . Completeness of reporting oligometastatic disease characteristics in the literature and influence on oligometastatic disease classification using the ESTRO/EORTC nomenclature. Int J Radiat Oncol Biol Phys. (2022) 114:587–95. doi: 10.1016/j.ijrobp.2022.06.067, PMID: 35738308

[8] Lievens Y Guckenberger M Gomez D Hoyer M Iyengar P Kindts I . Defining oligometastatic disease from a radiation oncology perspective: An ESTRO-ASTRO consensus document. Radiother Oncol. (2020) 148:157–66. doi: 10.1016/j.radonc.2020.04.003, PMID: 32388150

[9] Oprea-Lager DE MacLennan S Bjartell A Briganti A Burger IA de Jong I . European association of nuclear medicine focus 5: consensus on molecular imaging and theranostics in prostate cancer. Eur Urol. (2024) 85:49–60. doi: 10.1016/j.eururo.2023.09.003, PMID: 37743194

[10] Jadvar H Abreu AL Ballas LK Quinn DI . Oligometastatic prostate cancer: current status and future challenges. J Nucl Med. (2022) 63:1628–35. doi: 10.2967/jnumed.121.263124, PMID: 36319116 PMC9635685

[11] Palma DA Olson R Harrow S Correa RJM Schneiders F Haasbeek CJA . Stereotactic ablative radiotherapy for the comprehensive treatment of 4-10 oligometastatic tumors (SABR-COMET-10): study protocol for a randomized phase III trial. BMC Cancer. (2019) 19:816. doi: 10.1186/s12885-019-5977-6, PMID: 31426760 PMC6699121

[12] Ashram S Bahig H Barry A Blanchette D Celinksi A Chung P . Planning trade-offs for SABR in patients with 4 to 10 metastases: A substudy of the SABR-COMET-10 randomized trial. Int J Radiat Oncol Biol Phys. (2022) 114:1011–5. doi: 10.1016/j.ijrobp.2022.05.035, PMID: 35667527

[13] Guckenberger M Lievens Y Bouma AB Collette L Dekker A deSouza NM . Characterisation and classification of oligometastatic disease: a European Society for Radiotherapy and Oncology and European Organisation for Research and Treatment of Cancer consensus recommendation. Lancet Oncol. (2020) 21:e18–28. doi: 10.1016/s1470-2045(19)30718-1, PMID: 31908301

[14] Sutera PA Shetty AC Hakansson A Van der Eecken K Song Y Liu Y . Transcriptomic and clinical heterogeneity of metastatic disease timing within metastatic castration-sensitive prostate cancer. Ann Oncol. (2023) 34:605–14. doi: 10.1016/j.annonc.2023.04.515, PMID: 37164128 PMC10330666

[15] Mathew Thomas V Sayegh N Chigarira B Gebrael G Tripathi N Nussenzveig R . Differences in tumor gene expression profiles between *de novo* metastatic castration-sensitive prostate cancer and metastatic relapse after prior localized therapy. Eur Urol Oncol. (2024) 7:1462–8. doi: 10.1016/j.euo.2024.04.013, PMID: 38735779

[16] Deek MP van der Eecken K Phillips R Parikh NR Isaacsson Velho P Lotan TL . The mutational landscape of metastatic castration-sensitive prostate cancer: the spectrum theory revisited. Eur Urol. (2021) 80:632–40. doi: 10.1016/j.eururo.2020.12.040, PMID: 33419682 PMC10262980

[17] Sutera P Deek MP van der Eecken K Shetty AC Chang JH Hodges T . WNT pathway mutations in metachronous oligometastatic castration-sensitive prostate cancer. Int J Radiat Oncol Biol Phys. (2023) 115:1095–101. doi: 10.1016/j.ijrobp.2022.12.006, PMID: 36708787 PMC10443895

[18] Gillessen S Turco F Davis ID Efstathiou JA Fizazi K James ND . Management of patients with advanced prostate cancer. Report from the 2024 advanced prostate cancer consensus conference (APCCC). Eur Urol. (2025) 87:157–216. doi: 10.1016/j.eururo.2024.09.017, PMID: 39394013

[19] García-Zoghby L Lucas-Lucas C Amo-Salas M Soriano-Castrejón ÁM García-Vicente AM . Head-to-head comparison of [^18^F]F-choline and imaging of prostate-specific membrane antigen, using [^18^F]DCFPyL PET/CT, in patients with biochemical recurrence of prostate cancer. Curr Oncol. (2023) 30:6271–88. doi: 10.3390/curroncol30070464, PMID: 37504324 PMC10378109

[20] Tosoian JJ Gorin MA Ross AE Pienta KJ Tran PT Schaeffer EM . Oligometastatic prostate cancer: definitions, clinical outcomes, and treatment considerations. Nat Rev Urol. (2017) 14:15–25. doi: 10.1038/nrurol.2016.175, PMID: 27725639 PMC5808411

[21] Filippi L Bagni O Schillaci O . Digital PET/CT with ^18^F-FACBC in early castration-resistant prostate cancer: our preliminary results. Expert Rev Med Devices. (2022) 19:591–8. doi: 10.1080/17434440.2022.2117612, PMID: 36001041

[22] Amorim BJ Prabhu V Marco SS Gervais D Palmer WE Heidari P . Performance of ^18^F-fluciclovine PET/MR in the evaluation of osseous metastases from castration-resistant prostate cancer. Eur J Nucl Med Mol Imaging. (2020) 47:105–14. doi: 10.1007/s00259-019-04506-1, PMID: 31492992

[23] Shanmugasundaram R Saad J Heyworth A Wong V Pelecanos A Arianayagam M . Intra-individual comparison of prostate-specific membrane antigen positron emission tomography/computed tomography versus bone scan in detecting skeletal metastasis at prostate cancer diagnosis. BJU Int. (2024) 133 Suppl 3:25–32. doi: 10.1111/bju.16115, PMID: 37943964

[24] Quarta L Mazzone E Cannoletta D Stabile A Scuderi S Barletta F . Defining the optimal target-to-background ratio to identify positive lymph nodes in prostate cancer patients undergoing robot-assisted [^99m^Tc]Tc-PSMA radioguided surgery: updated results and ad interim analyses of a prospective phase II study. Eur J Nucl Med Mol Imaging. (2024) 51:3789–98. doi: 10.1007/s00259-024-06789-5, PMID: 38861182 PMC11445289

[25] Kuten J Dekalo S Mintz I Yossepowitch O Mano R Even-Sapir E . The significance of equivocal bone findings in staging PSMA imaging in the preoperative setting: validation of the PSMA-RADS version 1.0. EJNMMI Res. (2021) 11:3. doi: 10.1186/s13550-020-00745-8, PMID: 33409930 PMC7788112

[26] Mazzone E Cannoletta D Quarta L Chen DC Thomson A Barletta F . A comprehensive systematic review and meta-analysis of the role of prostate-specific membrane antigen positron emission tomography for prostate cancer diagnosis and primary staging before definitive treatment. Eur Urol. (2025) 87:654–71. doi: 10.1016/j.eururo.2025.03.003, PMID: 40155242

[27] Kleiburg F de Geus-Oei LF Spijkerman R Noortman WA van Velden FHP Manohar S . Baseline PSMA PET/CT parameters predict overall survival and treatment response in metastatic castration-resistant prostate cancer patients. Eur Radiol. (2025) 35:4223–32. doi: 10.1007/s00330-025-11360-3, PMID: 39843627 PMC12165979

[28] McHugh JK Bancroft EK Saunders E Brook MN McGrowder E Wakerell S . Assessment of a polygenic risk score in screening for prostate cancer. N Engl J Med. (2025) 392:1406–17. doi: 10.1056/nejmoa2407934, PMID: 40214032 PMC7617604

[29] Oishi T Hatakeyama S Tabata R Fujimori D Fukuda M Shinozaki T . Comparison of neoadjuvant chemohormonal therapy vs. extended pelvic lymph-node dissection in high-risk prostate cancer treated with robot-assisted radical prostatectomy. Sci Rep. (2023) 13:3436. doi: 10.1038/s41598-023-30627-7, PMID: 36859718 PMC9978020

[30] Xie X Zhang P Ran C Liu L Hu J Lei P . Global research status and hotspots of radiotherapy for prostate cancer: a bibliometric analysis based on Web of Science from 2010-2022. Front Oncol. (2023) 13:1135052. doi: 10.3389/fonc.2023.1135052, PMID: 37637069 PMC10450940

[31] Yanagisawa T Rajwa P Thibault C Gandaglia G Mori K Kawada T . Androgen receptor signaling inhibitors in addition to docetaxel with androgen deprivation therapy for metastatic hormone-sensitive prostate cancer: A systematic review and meta-analysis. Eur Urol. (2022) 82:584–98. doi: 10.1016/j.eururo.2022.08.002, PMID: 35995644

[32] Rogowski P Roach M 3rd Schmidt-Hegemann NS Trapp C von Bestenbostel R Shi R . Radiotherapy of oligometastatic prostate cancer: a systematic review. Radiat Oncol. (2021) 16:50. doi: 10.1186/s13014-021-01776-8, PMID: 33750437 PMC7941976

[33] Gillessen S Bossi A Davis ID de Bono J Fizazi K James ND . Management of patients with advanced prostate cancer. Part I: intermediate-/high-risk and locally advanced disease, biochemical relapse, and side effects of hormonal treatment: report of the advanced prostate cancer consensus conference 2022. Eur Urol. (2023) 83:267–93. doi: 10.1016/j.eururo.2022.11.002, PMID: 36494221 PMC7614721

[34] Cornford P van den Bergh RCN Briers E Van den Broeck T Cumberbatch MG De Santis M . EAU-EANM-ESTRO-ESUR-SIOG guidelines on prostate cancer. Part II-2020 update: treatment of relapsing and metastatic prostate cancer. Eur Urol. (2021) 79:263–82. doi: 10.1016/j.eururo.2020.09.046, PMID: 33039206

[35] Parker CC James ND Brawley CD Clarke NW Hoyle AP Ali A . Radiotherapy to the primary tumour for newly diagnosed, metastatic prostate cancer (STAMPEDE): a randomised controlled phase 3 trial. Lancet. (2018) 392:2353–66. doi: 10.1016/s0140-6736(18)32486-3, PMID: 30355464 PMC6269599

[36] Ham WS Park JS Jang WS Kim J . Radical prostatectomy versus radiotherapy as local therapy for primary tumors in patients with oligometastatic prostate cancer. Front Oncol. (2024) 14:1368926. doi: 10.3389/fonc.2024.1368926, PMID: 38544836 PMC10965631

[37] Heidenreich A Pfister D Porres D . Cytoreductive radical prostatectomy in patients with prostate cancer and low volume skeletal metastases: results of a feasibility and case-control study. J Urol. (2015) 193:832–8. doi: 10.1016/j.juro.2014.09.089, PMID: 25254935

[38] Rajwa P Robesti D Chaloupka M Zattoni F Giesen A Huebner NA . Outcomes of cytoreductive radical prostatectomy for oligometastatic prostate cancer on prostate-specific membrane antigen positron emission tomography: results of a multicenter European study. Eur Urol Oncol. (2024) 7:721–34. doi: 10.1016/j.euo.2023.09.006, PMID: 37845121

[39] Cheng B Li B Fu J Wang Q Luo T Li Z . Evaluating the effectiveness of cytoreductive surgery in oligometastatic prostate cancer: insights from quantitative analysis and retrospective cohort studies. Int J Surg. (2025) 111:122–34. doi: 10.1097/js9.0000000000001968, PMID: 39007913 PMC11745695

[40] Tang C Sherry AD Haymaker C Bathala T Liu S Fellman B . Addition of metastasis-directed therapy to intermittent hormone therapy for oligometastatic prostate cancer: the EXTEND phase 2 randomized clinical trial. JAMA Oncol. (2023) 9:825–34. doi: 10.1001/jamaoncol.2023.0161, PMID: 37022702 PMC10080407

[41] Marvaso G Corrao G Zaffaroni M Vincini MG Lorubbio C Gandini S . ADT with SBRT versus SBRT alone for hormone-sensitive oligorecurrent prostate cancer (RADIOSA): a randomised, open-label, phase 2 clinical trial. Lancet Oncol. (2025) 26:300–11. doi: 10.1016/s1470-2045(24)00730-7, PMID: 40049196

[42] Decaestecker K De Meerleer G Ameye F Fonteyne V Lambert B Joniau S . Surveillance or metastasis-directed Therapy for OligoMetastatic Prostate cancer recurrence (STOMP): study protocol for a randomized phase II trial. BMC Cancer. (2014) 14:671. doi: 10.1186/1471-2407-14-671, PMID: 25223986 PMC4175227

[43] Ost P Reynders D Decaestecker K Fonteyne V Lumen N De Bruycker A . Surveillance or metastasis-directed therapy for oligometastatic prostate cancer recurrence: A prospective, randomized, multicenter phase II trial. J Clin Oncol. (2018) 36:446–53. doi: 10.1200/jco.2017.75.4853, PMID: 29240541

[44] Phillips R Shi WY Deek M Radwan N Lim SJ Antonarakis ES . Outcomes of observation vs stereotactic ablative radiation for oligometastatic prostate cancer: the ORIOLE phase 2 randomized clinical trial. JAMA Oncol. (2020) 6:650–9. doi: 10.1001/jamaoncol.2020.0147, PMID: 32215577 PMC7225913

[45] See AW Conway P Frydenberg M Haxhimolla H Costello AJ Moon D . Five-year outcomes of fractionated stereotactic body radiotherapy for oligometastatic prostate cancer from the TRANSFORM phase II trial. Int J Cancer. (2024) 155:1248–56. doi: 10.1002/ijc.35052, PMID: 38898626

[46] Ferriero M Prata F Mastroianni R De Nunzio C Tema G Tuderti G . The impact of locoregional treatments for metastatic castration resistant prostate cancer on disease progression: real life experience from a multicenter cohort. Prostate Cancer Prostatic Dis. (2024) 27:89–94. doi: 10.1038/s41391-022-00623-5, PMID: 36460734

[47] Wang JH Sherry AD Bazyar S Sutera P Radwan N Phillips RM . Outcomes of radium-223 and stereotactic ablative radiotherapy versus stereotactic ablative radiotherapy for oligometastatic prostate cancers: the RAVENS phase II randomized trial. J Clin Oncol. (2025) 43:2059–68. doi: 10.1200/jco-25-00131, PMID: 40334149 PMC12169860

[48] Grkovski M O’Donoghue JA Imber BS Andl G Tu C Lafontaine D . Lesion dosimetry for [^177^Lu]Lu-PSMA-617 radiopharmaceutical therapy combined with stereotactic body radiotherapy in patients with oligometastatic castration-sensitive prostate cancer. J Nucl Med. (2023) 64:1779–87. doi: 10.2967/jnumed.123.265763, PMID: 37652541 PMC10626375

[49] Potkrajcic V Mueller AC Frey B Gani C Zips D Hoffmann R . Dose-escalated radiotherapy with simultaneous integrated boost for bone metastases in selected patients with assumed favourable prognosis. Radiol Oncol. (2022) 56:515–24. doi: 10.2478/raon-2022-0053, PMID: 36503710 PMC9784373

[50] Deodato F Pezzulla D Cilla S Ferro M Giannini R Romano C . Volumetric intensity-modulated arc stereotactic radiosurgery boost in oligometastatic patients with spine metastases: a dose-escalation study. Clin Oncol (R Coll Radiol). (2023) 35:e30–9. doi: 10.1016/j.clon.2022.09.045, PMID: 36207236

[51] Ferriero M Prata F Anceschi U Astore S Bove AM Brassetti A . Oncological outcomes of patients with high-volume mCRPC: results from a longitudinal real-life multicenter cohort. Cancers (Basel). (2023) 15:4809. doi: 10.3390/cancers15194809, PMID: 37835503 PMC10571997

[52] Baron D Pasquier D Pace-Loscos T Vandendorpe B Schiappa R Ortholan C . Systemic therapy escalation after stereotactic body radiation therapy for oligometastatic hormone-sensitive prostate cancer. Clin Transl Radiat Oncol. (2023) 43:100673. doi: 10.1016/j.ctro.2023.100673, PMID: 37701481 PMC10493250

[53] Cuccia F Tamburo M Piras A Mortellaro G Iudica A Daidone A . Stereotactic body radiotherapy for lymph-nodal oligometastatic prostate cancer: A multicenter retrospective experience. Med (Kaunas). (2023) 59:1442. doi: 10.3390/medicina59081442, PMID: 37629732 PMC10456704

[54] Boevé LMS Hulshof MCCM Vis AN Zwinderman AH Twisk JWR Witjes WPJ . Effect on survival of androgen deprivation therapy alone compared to androgen deprivation therapy combined with concurrent radiation therapy to the prostate in patients with primary bone metastatic prostate cancer in a prospective randomised clinical trial: data from the HORRAD trial. Eur Urol. (2019) 75:410–8. doi: 10.1016/j.eururo.2018.09.008, PMID: 30266309

[55] Armstrong AJ Iguchi T Azad AA Villers A Alekseev B Petrylak DP . The efficacy of enzalutamide plus androgen deprivation therapy in oligometastatic hormone-sensitive prostate cancer: A *post hoc* analysis of ARCHES. Eur Urol. (2023) 84:229–41. doi: 10.1016/j.eururo.2023.04.002, PMID: 37179240

[56] Armstrong AJ Szmulewitz RZ Petrylak DP Holzbeierlein J Villers A Azad A . ARCHES: A randomized, phase III study of androgen deprivation therapy with enzalutamide or placebo in men with metastatic hormone-sensitive prostate cancer. J Clin Oncol. (2019) 37:2974–86. doi: 10.1200/jco.19.00799, PMID: 31329516 PMC6839905

[57] Saad F Vjaters E Shore N Olmos D Xing N Pereira de Santana Gomes AJ . Darolutamide in combination with androgen-deprivation therapy in patients with metastatic hormone-sensitive prostate cancer from the phase III ARANOTE trial. J Clin Oncol. (2024) 42:4271–81. doi: 10.1200/jco-24-01798, PMID: 39279580 PMC11654448

[58] Chi KN Chowdhury S Bjartell A Chung BH Pereira de Santana Gomes AJ Given R . Apalutamide in patients with metastatic castration-sensitive prostate cancer: final survival analysis of the randomized, double-blind, phase III TITAN study. J Clin Oncol. (2021) 39:2294–303. doi: 10.1200/jco.20.03488, PMID: 33914595

[59] Chi KN Agarwal N Bjartell A Chung BH Pereira de Santana Gomes AJ Given R . Apalutamide for metastatic, castration-sensitive prostate cancer. N Engl J Med. (2019) 381:13–24. doi: 10.1056/nejmoa1903307, PMID: 31150574

[60] Sweeney CJ Martin AJ Stockler MR Begbie S Cheung L Chi KN . Testosterone suppression plus enzalutamide versus testosterone suppression plus standard antiandrogen therapy for metastatic hormone-sensitive prostate cancer (ENZAMET): an international, open-label, randomised, phase 3 trial. Lancet Oncol. (2023) 24:323–34. doi: 10.1016/s1470-2045(23)00063-3, PMID: 36990608

[61] Agarwal N Tangen CM Hussain MHA Gupta S Plets M Lara PN . Orteronel for metastatic hormone-sensitive prostate cancer: A multicenter, randomized, open-label phase III trial (SWOG-1216). J Clin Oncol. (2022) 40:3301–9. doi: 10.1200/jco.21.02517, PMID: 35446628 PMC9553390

[62] Fizazi K Tran N Fein L Matsubara N Rodriguez-Antolin A Alekseev BY . Abiraterone acetate plus prednisone in patients with newly diagnosed high-risk metastatic castration-sensitive prostate cancer (LATITUDE): final overall survival analysis of a randomised, double-blind, phase 3 trial. Lancet Oncol. (2019) 20:686–700. doi: 10.1016/s1470-2045(19)30082-8, PMID: 30987939

[63] Fizazi K Tran N Fein L Matsubara N Rodriguez-Antolin A Alekseev BY . Abiraterone plus prednisone in metastatic, castration-sensitive prostate cancer. N Engl J Med. (2017) 377:352–60. doi: 10.1056/nejmoa1704174, PMID: 28578607

[64] Kyriakopoulos CE Chen YH Carducci MA Liu G Jarrard DF Hahn NM . Chemohormonal therapy in metastatic hormone-sensitive prostate cancer: long-term survival analysis of the randomized phase III E3805 CHAARTED trial. J Clin Oncol. (2018) 36:1080–7. doi: 10.1200/jco.2017.75.3657, PMID: 29384722 PMC5891129

[65] Gravis G Fizazi K Joly F Oudard S Priou F Esterni B . Androgen-deprivation therapy alone or with docetaxel in non-castrate metastatic prostate cancer (GETUG-AFU 15): a randomised, open-label, phase 3 trial. Lancet Oncol. (2013) 14:149–58. doi: 10.1016/s1470-2045(12)70560-0, PMID: 23306100

[66] Deek MP Sutera P Jing Y Gao R Rothman E Day H . Multi-institutional analysis of metastasis-directed therapy with or without androgen deprivation therapy in oligometastatic castration-sensitive prostate cancer. Eur Urol Oncol. (2024) 7:1403–10. doi: 10.1016/j.euo.2024.03.010, PMID: 38570239

[67] Conde-Moreno AJ López-Campos F Hervás A Morillo V Méndez A Puertas MDM . A phase II trial of stereotactic body radiation therapy and androgen deprivation for oligometastases in prostate cancer (SBRT-SG 05). Pract Radiat Oncol. (2024) 14:e344–52. doi: 10.1016/j.prro.2024.04.022, PMID: 38944806

[68] Fizazi K Foulon S Carles J Roubaud G McDermott R Fléchon A . Abiraterone plus prednisone added to androgen deprivation therapy and docetaxel in *de novo* metastatic castration-sensitive prostate cancer (PEACE-1): a multicentre, open-label, randomised, phase 3 study with a 2 × 2 factorial design. Lancet. (2022) 399:1695–707. doi: 10.1016/s0140-6736(22)00367-1, PMID: 35405085

[69] Hussain M Tombal B Saad F Fizazi K Sternberg CN Crawford ED . Darolutamide plus androgen-deprivation therapy and docetaxel in metastatic hormone-sensitive prostate cancer by disease volume and risk subgroups in the phase III ARASENS trial. J Clin Oncol. (2023) 41:3595–607. doi: 10.1200/jco.23.00041, PMID: 36795843

[70] Turner PG Jain S Cole A Grey A Mitchell D Prise KM . Toxicity and efficacy of concurrent androgen deprivation therapy, pelvic radiotherapy, and radium-223 in patients with *de novo* metastatic hormone-sensitive prostate cancer. Clin Cancer Res. (2021) 27:4549–56. doi: 10.1158/1078-0432.ccr-21-0685, PMID: 34187853

[71] Nickols NG Tsai S Kane N Tran S Ghayouri L Diaz-Perez S . Systemic and tumor-directed therapy for oligometastatic prostate cancer: the SOLAR phase 2 trial in *de novo* oligometastatic prostate cancer. Eur Urol. (2024) 86:190–3. doi: 10.1016/j.eururo.2024.02.008, PMID: 38490853 PMC12363338

[72] Nikitas J Rettig M Shen J Reiter R Lee A Steinberg ML . Systemic and tumor-directed therapy for oligorecurrent metastatic prostate cancer (SATURN): primary endpoint results from a phase 2 clinical trial. Eur Urol. (2024) 85:517–20. doi: 10.1016/j.eururo.2024.01.021, PMID: 38494380 PMC11386258

[73] O’Shaughnessy MJ McBride SM Vargas HA Touijer KA Morris MJ Danila DC . A pilot study of a multimodal treatment paradigm to accelerate drug evaluations in early-stage metastatic prostate cancer. Urology. (2017) 102:164–72. doi: 10.1016/j.urology.2016.10.044, PMID: 27888148 PMC5468169

[74] Reyes DK Rowe SP Schaeffer EM Allaf ME Ross AE Pavlovich CP . Multidisciplinary total eradication therapy (TET) in men with newly diagnosed oligometastatic prostate cancer. Med Oncol. (2020) 37:60. doi: 10.1007/s12032-020-01385-7, PMID: 32524295 PMC7286864

[75] Deek MP van der Eecken K Sutera P Deek RA Fonteyne V Mendes AA . Long-term outcomes and genetic predictors of response to metastasis-directed therapy versus observation in oligometastatic prostate cancer: analysis of STOMP and ORIOLE trials. J Clin Oncol. (2022) 40:3377–82. doi: 10.1200/jco.22.00644, PMID: 36001857 PMC10166371

[76] Armstrong AJ Azad AA Iguchi T Szmulewitz RZ Petrylak DP Holzbeierlein J . Improved survival with enzalutamide in patients with metastatic hormone-sensitive prostate cancer. J Clin Oncol. (2022) 40:1616–22. doi: 10.1200/jco.22.00193, PMID: 35420921 PMC9113211

[77] Agarwal N McQuarrie K Bjartell A Chowdhury S Pereira de Santana Gomes AJ Chung BH . Health-related quality of life after apalutamide treatment in patients with metastatic castration-sensitive prostate cancer (TITAN): a randomised, placebo-controlled, phase 3 study. Lancet Oncol. (2019) 20:1518–30. doi: 10.1016/s1470-2045(19)30620-5, PMID: 31578173

[78] Gebrael G Jo Y Swami U Plets M Hage Chehade C Narang A . Bone pain and survival among patients with metastatic, hormone-sensitive prostate cancer: A secondary analysis of the SWOG-1216 trial. JAMA Netw Open. (2024) 7:e2419966. doi: 10.1001/jamanetworkopen.2024.19966, PMID: 38980676 PMC11234233

[79] Chi KN Protheroe A Rodríguez-Antolín A Facchini G Suttman H Matsubara N . Patient-reported outcomes following abiraterone acetate plus prednisone added to androgen deprivation therapy in patients with newly diagnosed metastatic castration-naive prostate cancer (LATITUDE): an international, randomised phase 3 trial. Lancet Oncol. (2018) 19:194–206. doi: 10.1016/s1470-2045(17)30911-7, PMID: 29326030

[80] Matsubara N Chi KN Özgüroğlu M Rodriguez-Antolin A Feyerabend S Fein L . Correlation of prostate-specific antigen kinetics with overall survival and radiological progression-free survival in metastatic castration-sensitive prostate cancer treated with abiraterone acetate plus prednisone or placebos added to androgen deprivation therapy: *post hoc* analysis of phase 3 LATITUDE study. Eur Urol. (2020) 77:494–500. doi: 10.1016/j.eururo.2019.11.021, PMID: 31843335

[81] Bossi A Foulon S Maldonado X Sargos P MacDermott R Kelly P . Efficacy and safety of prostate radiotherapy in *de novo* metastatic castration-sensitive prostate cancer (PEACE-1): a multicentre, open-label, randomised, phase 3 study with a 2 × 2 factorial design. Lancet. (2024) 404:2065–76. doi: 10.1016/s0140-6736(24)01865-8, PMID: 39580202

[82] Smith MR Hussain M Saad F Fizazi K Sternberg CN Crawford ED . Darolutamide and survival in metastatic, hormone-sensitive prostate cancer. N Engl J Med. (2022) 386:1132–42. doi: 10.1056/nejmoa2119115, PMID: 35179323 PMC9844551

[83] Menges D Yebyo HG Sivec-Muniz S Haile SR Barbier MC Tomonaga Y . Treatments for metastatic hormone-sensitive prostate cancer: systematic review, network meta-analysis, and benefit-harm assessment. Eur Urol Oncol. (2022) 5:605–16. doi: 10.1016/j.euo.2022.04.007, PMID: 35599144

[84] Parker CC Kynaston H Cook AD Clarke NW Catton CN Cross WR . Duration of androgen deprivation therapy with postoperative radiotherapy for prostate cancer: a comparison of long-course versus short-course androgen deprivation therapy in the RADICALS-HD randomised trial. Lancet. (2024) 403:2416–25. doi: 10.1016/s0140-6736(24)00549-x, PMID: 38763153 PMC7616389

[85] Juarez Casillas JE Nikitas J Rettig MB Reiter RE Lee A Steinberg ML . Pooled analysis of the SOLAR and SATURN clinical trials comparing progression of synchronous versus metachronous prostate-specific membrane antigen-defined oligometastatic prostate cancer following systemic and tumor-directed therapy. Eur Urol Oncol. (2025) 8:893–8. doi: 10.1016/j.euo.2025.05.027, PMID: 40541485 PMC12311854

[86] Chang Y Zhao X Xiao Y Yan S Xu W Wang Y . Neoadjuvant radiohormonal therapy for oligo-metastatic prostate cancer: safety and efficacy outcomes from an open-label, dose-escalation, single-center, phase I/II clinical trial. Front Med. (2023) 17:231–9. doi: 10.1007/s11684-022-0939-9, PMID: 36580231

[87] Li X Han Z Ai J . Synergistic targeting strategies for prostate cancer. Nat Rev Urol. (2025) 22:645–71. doi: 10.1038/s41585-025-01042-6, PMID: 40394240

[88] Francolini G Porreca A Facchini G Santini D Bruni A Simoni N . PERSIAN trial (NCT05717660): an ongoing randomized trial testing androgen deprivation therapy, apalutamide and stereotactic body radiotherapy. An alternative “triplet” for oligometastatic hormone sensitive prostate cancer patients. Med Oncol. (2023) 41:39. doi: 10.1007/s12032-023-02268-3, PMID: 38157111

[89] Li X Xi H Cheng X Yu Y Zhang C Wang G . Assessment of oligometastasis status of prostate cancer following combined robot-assisted radical prostatectomy and androgen deprivation versus androgen deprivation therapy alone using PSA percentage decline rate. Front Endocrinol (Lausanne). (2023) 14:1123934. doi: 10.3389/fendo.2023.1123934, PMID: 36843605 PMC9951113

[90] Saad F Hussain MHA Tombal B Fizazi K Sternberg CN Crawford ED . Deep and durable prostate-specific antigen response to darolutamide with androgen deprivation therapy and docetaxel, and association with clinical outcomes for patients with high- or low-volume metastatic hormone-sensitive prostate cancer: analyses of the randomized phase 3 ARASENS study. Eur Urol. (2024) 86:329–39. doi: 10.1016/j.eururo.2024.03.036, PMID: 38644146

[91] Harshman LC Chen YH Liu G Carducci MA Jarrard D Dreicer R . Seven-month prostate-specific antigen is prognostic in metastatic hormone-sensitive prostate cancer treated with androgen deprivation with or without docetaxel. J Clin Oncol. (2018) 36:376–82. doi: 10.1200/jco.2017.75.3921, PMID: 29261442 PMC5805480

[92] Ali A Hoyle A Haran ÁM Brawley CD Cook A Amos C . Association of bone metastatic burden with survival benefit from prostate radiotherapy in patients with newly diagnosed metastatic prostate cancer: A secondary analysis of a randomized clinical trial. JAMA Oncol. (2021) 7:555–63. doi: 10.1001/jamaoncol.2020.7857, PMID: 33599706 PMC7893550

[93] Jiménez N Garcia de Herreros M Reig Ò Marín-Aguilera M Aversa C Ferrer-Mileo L . Development and independent validation of a prognostic gene expression signature based on RB1, PTEN, and TP53 in metastatic hormone-sensitive prostate cancer patients. Eur Urol Oncol. (2024) 7:954–64. doi: 10.1016/j.euo.2023.12.012, PMID: 38429210

[94] Hearn JWD Sweeney CJ Almassi N Reichard CA Reddy CA Li H . HSD3B1 genotype and clinical outcomes in metastatic castration-sensitive prostate cancer. JAMA Oncol. (2020) 6:e196496. doi: 10.1001/jamaoncol.2019.6496, PMID: 32053149 PMC7042830

[95] Hamid AA Huang HC Wang V Chen YH Feng F Den R . Transcriptional profiling of primary prostate tumor in metastatic hormone-sensitive prostate cancer and association with clinical outcomes: correlative analysis of the E3805 CHAARTED trial. Ann Oncol. (2021) 32:1157–66. doi: 10.1016/j.annonc.2021.06.003, PMID: 34129855 PMC8463957

[96] Ross AE Iwata KK Elsouda D Hairston J Russell D Davicioni E . Transcriptome-based prognostic and predictive biomarker analysis of ENACT: A randomized controlled trial of enzalutamide in men undergoing active surveillance. JCO Precis Oncol. (2024) 8:e2300603. doi: 10.1200/po.23.00603, PMID: 38635932 PMC11161222

[97] Kohli M Tan W Zheng T Wang A Montesinos C Wong C . Clinical and genomic insights into circulating tumor DNA-based alterations across the spectrum of metastatic hormone-sensitive and castrate-resistant prostate cancer. EBioMedicine. (2020) 54:102728. doi: 10.1016/j.ebiom.2020.102728, PMID: 32268276 PMC7186589

[98] Du X Fei X Wang J Dong Y Fan L Yang B . Early serial circulating tumor DNA sequencing predicts the efficacy of chemohormonal therapy in patients with metastatic hormone-sensitive prostate cancer. Transl Oncol. (2023) 34:101701. doi: 10.1016/j.tranon.2023.101701, PMID: 37247504 PMC10236461

[99] Andrews JR Kim Y Horjeti E Arafa A Gunn H De Bruycker A . PSMA+ Extracellular vesicles are a biomarker for SABR in oligorecurrent prostate cancer: analysis from the STOMP-like and ORIOLE trial cohorts. Clin Cancer Res. (2025) 31:1142–9. doi: 10.1158/1078-0432.ccr-24-3027, PMID: 39820657 PMC11911805

[100] Lin HM Scheinberg T Portman N Kim RMN Mellor R Huynh K . Association of the circulating lipid panel, PCPro, with clinical outcomes in metastatic hormone-sensitive prostate cancer: post hoc analysis of the ENZAMET phase III randomised trial (ANZUP 1304). Ann Oncol. (2025) 36:1068–77. doi: 10.1016/j.annonc.2025.05.529, PMID: 40403846

[101] Zaffaroni M Vincini MG Corrao G Lorubbio C Repetti I Mastroleo F . Investigating nutritional and inflammatory status as predictive biomarkers in oligoreccurent prostate cancer-A RADIOSA trial preliminary analysis. Nutrients. (2023) 15:4583. doi: 10.3390/nu15214583, PMID: 37960236 PMC10647217

[102] Wang JH Deek MP Mendes AA Song Y Shetty A Bazyar S . Validation of an artificial intelligence-based prognostic biomarker in patients with oligometastatic Castration-Sensitive prostate cancer. Radiother Oncol. (2025) 202:110618. doi: 10.1016/j.radonc.2024.110618, PMID: 39510141 PMC11663099

[103] Gebrael G Sayegh N Hage Chehade C Jo Y Narang A Chigarira B . Genomic biomarkers of survival in patients with metastatic hormone-sensitive prostate cancer undergoing intensified androgen deprivation therapy. Prostate Cancer Prostatic Dis. (2025) 28:887–93. doi: 10.1038/s41391-025-00936-1, PMID: 39885371

[104] Garcia de Herreros M Jiménez N Rodríguez-Carunchio L Lillo E Marín-Aguilera M Ferrer-Mileo L . Prognostic expression signature of RB1, PTEN, and TP53 genes in patients with metastatic hormone-sensitive prostate cancer treated with androgen receptor pathway inhibitors. Eur Urol Open Sci. (2024) 70:86–90. doi: 10.1016/j.euros.2024.10.008, PMID: 39502104 PMC11536031

[105] Spohn SKB Draulans C Kishan AU Spratt D Ross A Maurer T . Genomic classifiers in personalized prostate cancer radiation therapy approaches: A systematic review and future perspectives based on international consensus. Int J Radiat Oncol Biol Phys. (2023) 116:503–20. doi: 10.1016/j.ijrobp.2022.12.038, PMID: 36596346

[106] Ruiz-Vico M Wetterskog D Orlando F Thakali S Wingate A Jayaram A . Liquid biopsy in progressing prostate cancer patients starting docetaxel with or without enzalutamide: A biomarker study of the PRESIDE phase 3b trial. Eur Urol Oncol. (2025) 8:135–44. doi: 10.1016/j.euo.2024.08.006, PMID: 39261236

[107] Knutson TP Luo B Kobilka A Lyman J Guo S Munro SA . AR alterations inform circulating tumor DNA detection in metastatic castration resistant prostate cancer patients. Nat Commun. (2024) 15:10648. doi: 10.1038/s41467-024-54847-1, PMID: 39663356 PMC11634963

[108] Sutera P Song Y Shetty AC English K van der Eecken K Guler OC . Genomic determinants associated with modes of progression and patterns of failure in metachronous oligometastatic castration-sensitive prostate cancer. Eur Urol Oncol. (2025) 8:111–8. doi: 10.1016/j.euo.2024.05.011, PMID: 38862340

[109] Swami U Graf RP Nussenzveig RH Fisher V Tukachinsky H Schrock AB . SPOP mutations as a predictive biomarker for androgen receptor axis-targeted therapy in *de novo* metastatic castration-sensitive prostate cancer. Clin Cancer Res. (2022) 28:4917–25. doi: 10.1158/1078-0432.ccr-22-2228, PMID: 36088616

[110] Zhou J Lai Y Peng S Tang C Chen Y Li L . Comprehensive analysis of TP53 and SPOP mutations and their impact on survival in metastatic prostate cancer. Front Oncol. (2022) 12:957404. doi: 10.3389/fonc.2022.957404, PMID: 36119488 PMC9471084

[111] Urabe F Tashiro K Muramoto K Yanagisawa T Katsumi K Takahashi H . Locations of metastases in and oncological outcomes of patients with metastatic castration-sensitive prostate cancer: Real-world data from a multicenter study. Urol Oncol. (2025) 43:336.e1–336.e11. doi: 10.1016/j.urolonc.2025.02.007, PMID: 40023743

